# Supplementary material for: Reduced Chitosan as a Strategy for Removing Copper Ions from Water
Source: Molecules. 2023 May 16;28(10):4110. doi: 10.3390/molecules28104110 (PMC10224156; doi:10.3390/molecules28104110)
Supplement: Supplementary file 1 [file molecules-28-04110-s001.zip › molecules-2374392-supplementary.pdf]

molecules

MDPI

Supporting Information

Reduced Chitosan as a Strategy for Removing Copper Ions from Water

Pedro M. C. Matias [1], Joana F. M. Sousa [1], Eva F. Bernardino [1], João P. Vareda [2], Luisa Durães [2], Paulo E. Abreu [1], Jorge M. C. Marques [1], Dina Murtinho [1] and Artur J. M. Valente [1,*]

[1] University of Coimbra, CQC-IMS, Department of Chemistry, Rua Larga, 3004-535 Coimbra, Portugal; petermatias1998@gmail.com (P.M.C.M.); uc2011141774@student.uc.pt (J.F.M.S.); eva.fbernardino@gmail.com (E.F.B.); qtabreu@ci.uc.pt (P.E.A.); qtmarque@ci.uc.pt (J.M.C.M.); dmurtinho@ci.uc.pt (D.M.)

[2] University of Coimbra, CIEPQPF, Department of Chemical Engineering, Rua Sílvio Lima, 3030-790 Coimbra, Portugal; jvareda@eq.uc.pt (J.P.V.); luisa@eq.uc.pt (L.D.)

* Correspondence: avalente@ci.uc.pt

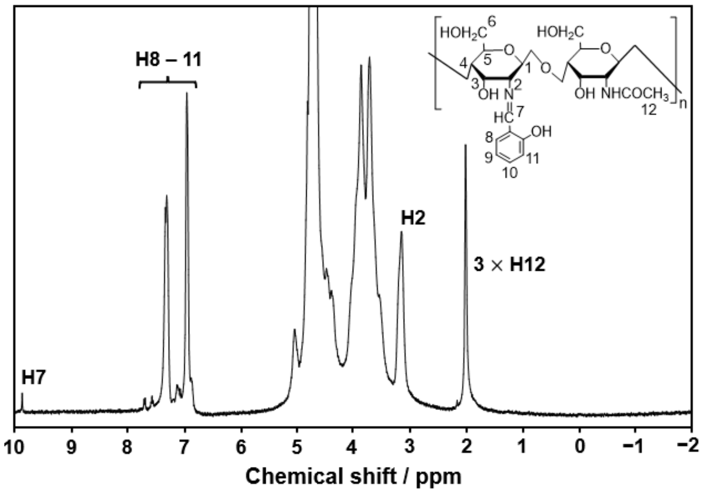

Figure S1. ^{1}H-RMN spectrum of RCD3 (7.5 mg mL^{-1}) in DCl:D_2O (1%, v/v).

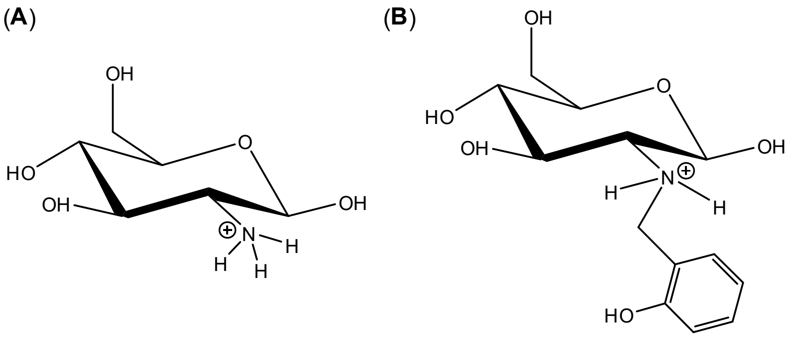

Figure S2. (**A**) Protonated monomer of chitosan (Chit+) and (**B**) Protonated unit of functionalized chitosan (RCD+).

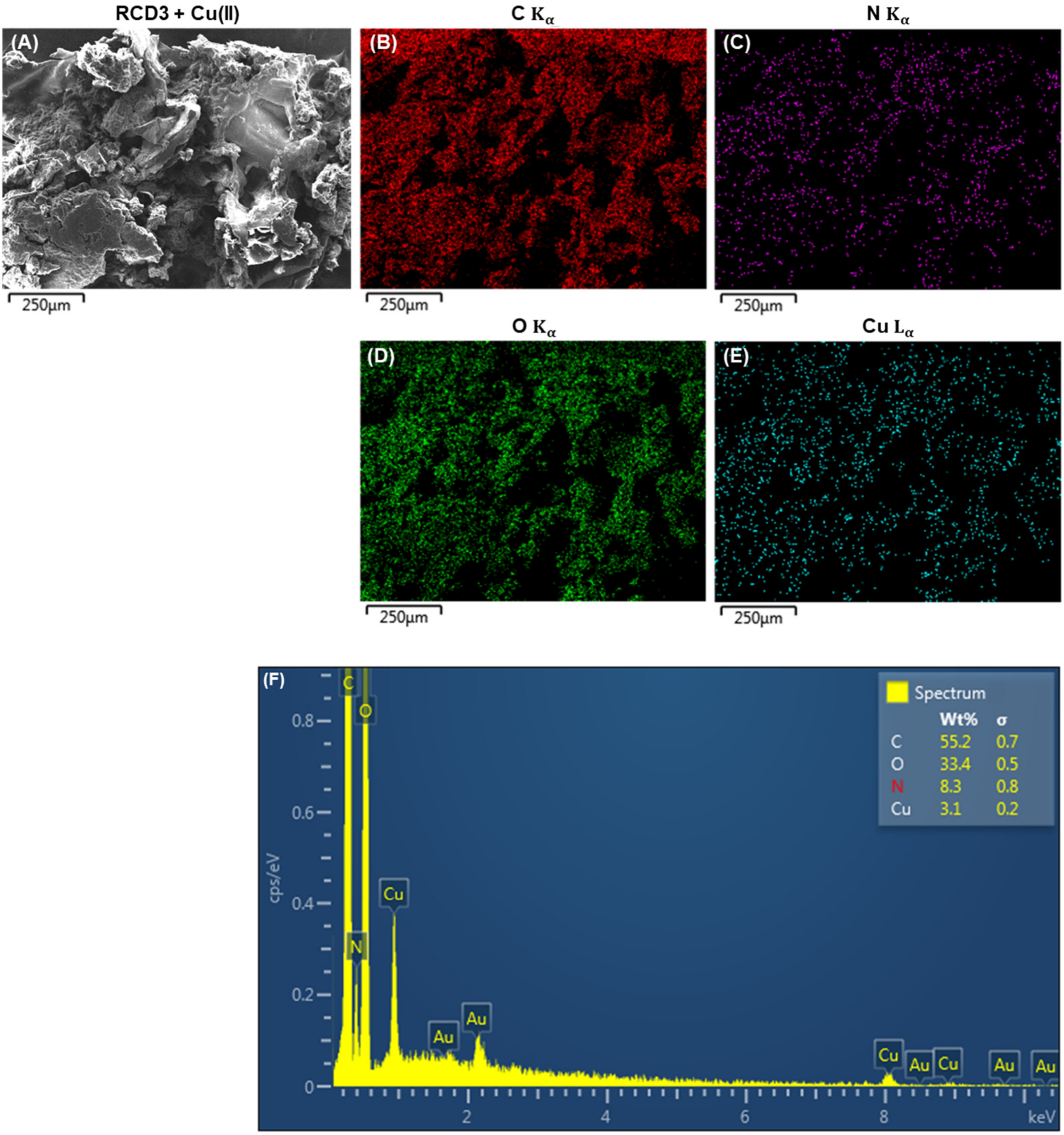

Figure S3. (**A**) SEM image of RCD3 immersed in Cu(II) aqueous solution for 24 hours; (**B**)-(**E**) corresponding carbon, nitrogen, oxygen and copper distributions, respectively, obtained by polymer mapping by EDS. (**F**) EDS spectrum showing the major components of RCD3 after Cu(II) adsorption.

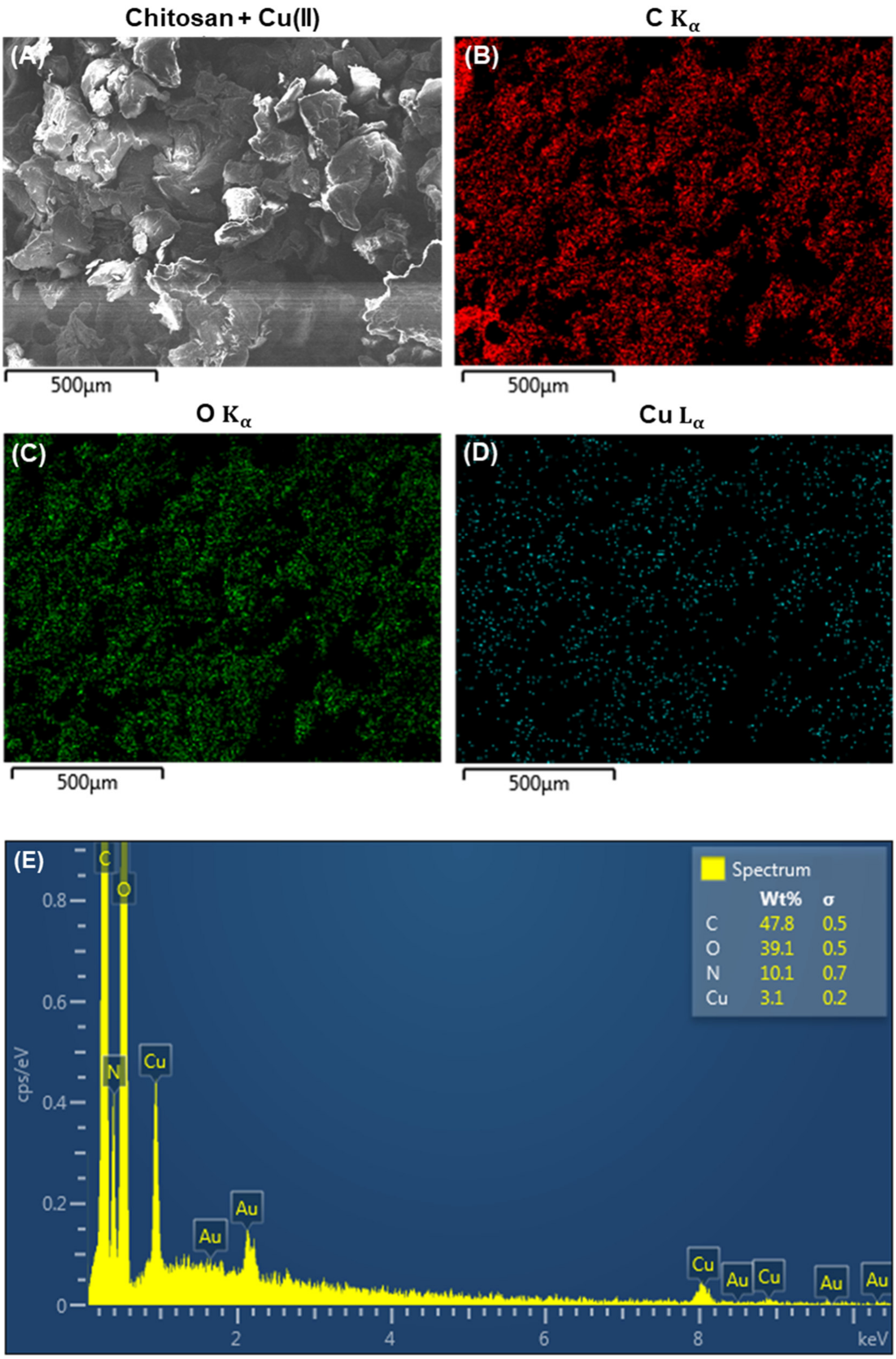

Figure S4. (**A**) SEM image of chitosan immersed in Cu(II) aqueous solution for 24 hours; (**B**)-(**D**) corresponding carbon, oxygen and copper distributions, respectively, obtained by polymer mapping by EDS. (**E**) EDS spectrum showing the major components of chitosan after Cu(II) adsorption.

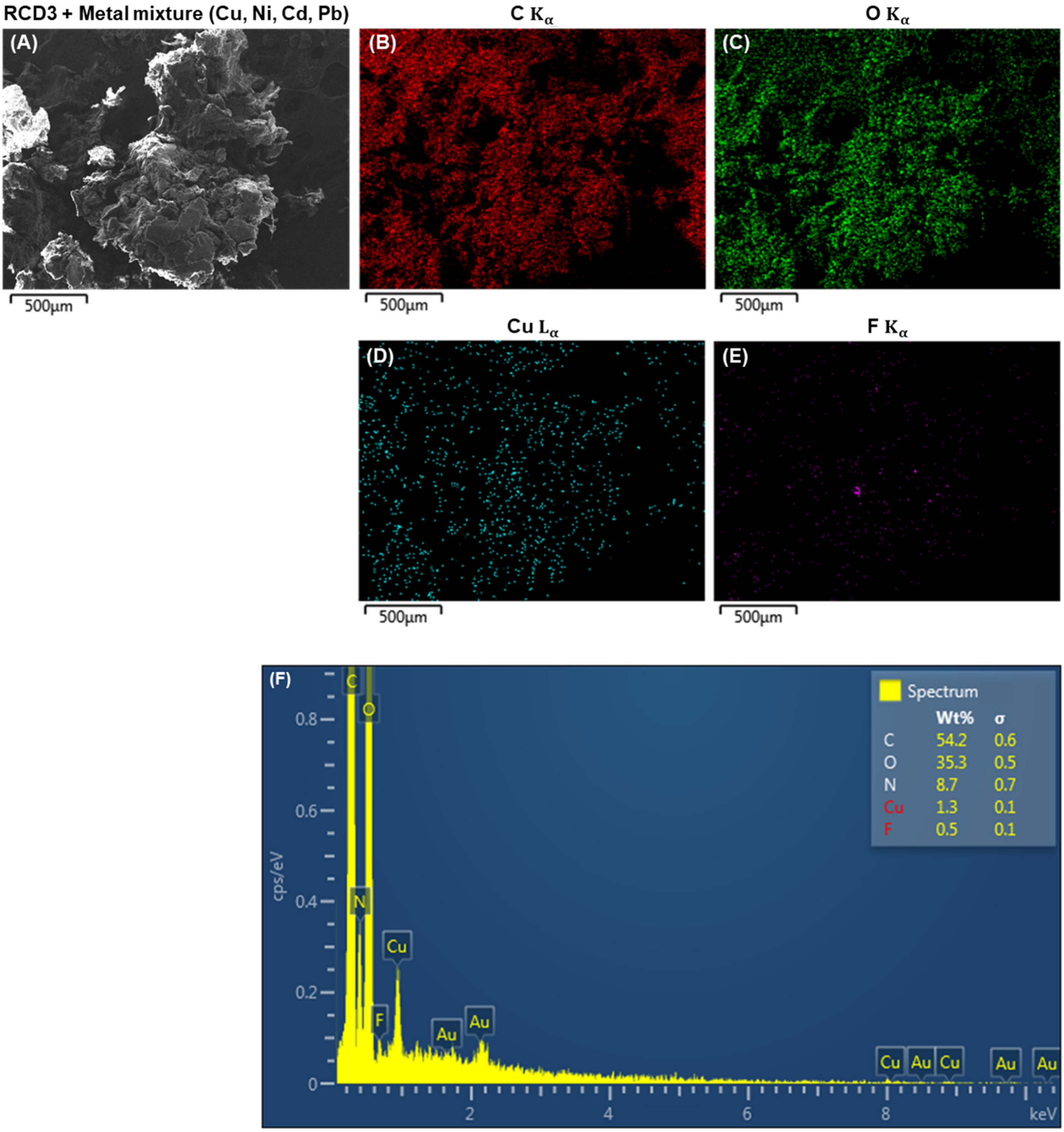

Figure S5. (**A**) SEM image of RCD3 immersed in an aqueous solution containing Cu(II), Ni(II), Cd(II), Pb(II) ions, for 24 hours; (**B**)-(**E**) corresponding carbon, oxygen, copper and fluorine distributions, respectively, obtained by polymer mapping by EDS. (**F**) EDS spectrum showing the major components of RCD3 after metal mixture adsorption (residual content indicated as fluorine can correspond to adsorbed nickel).

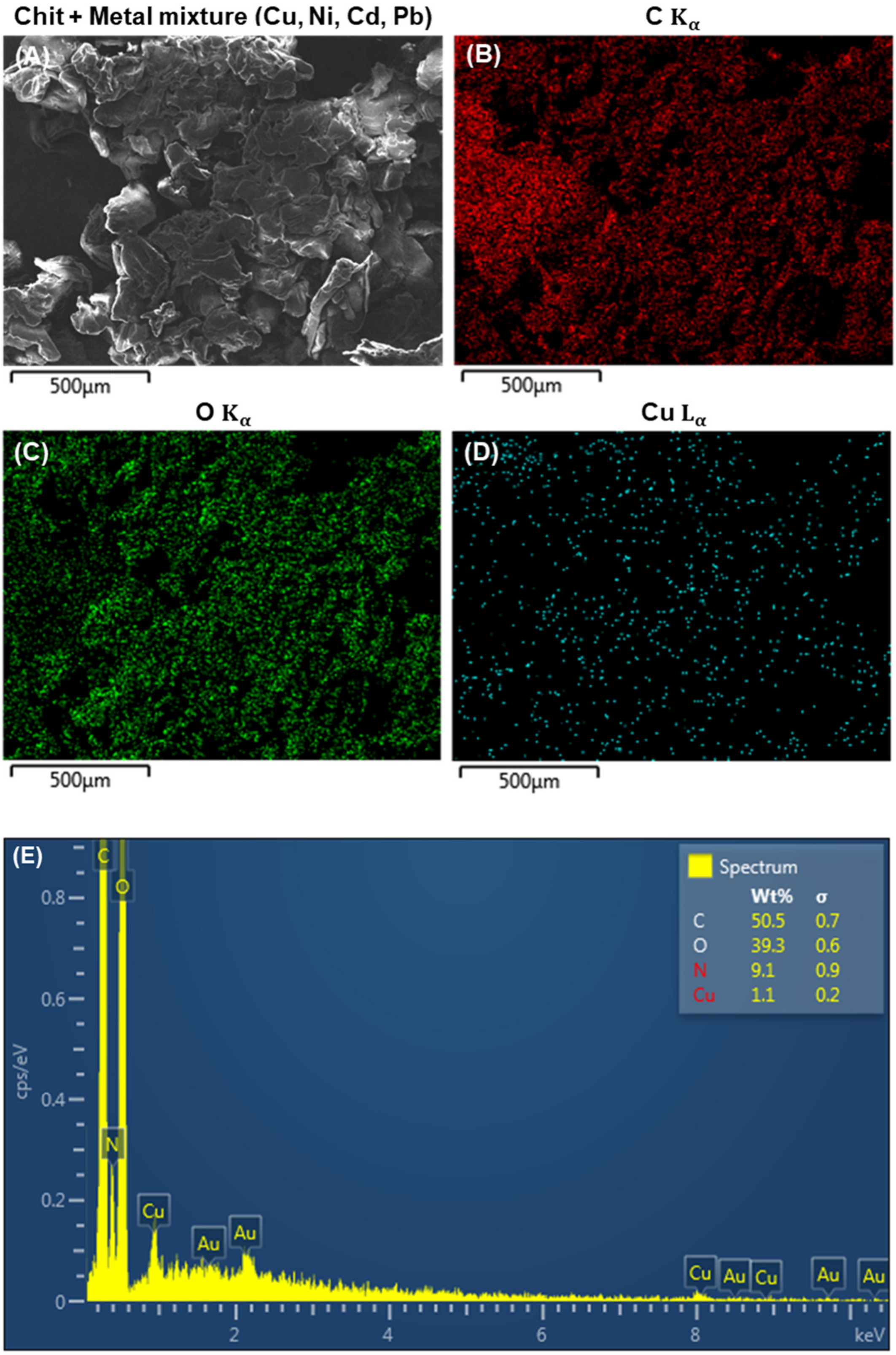

Figure S6. (**A**) SEM image of chitosan immersed in an aqueous solution containing Cu(II), Ni(II), Cd(II), Pb(II) ions, for 24 hours; (**B**)-(**D**) corresponding carbon, oxygen and copper distributions, respectively, obtained by polymer mapping by EDS. (**E**) EDS spectrum showing the major components of chitosan after metal mixture adsorption.

(**A**) (**B**) (**C**) (**D**)

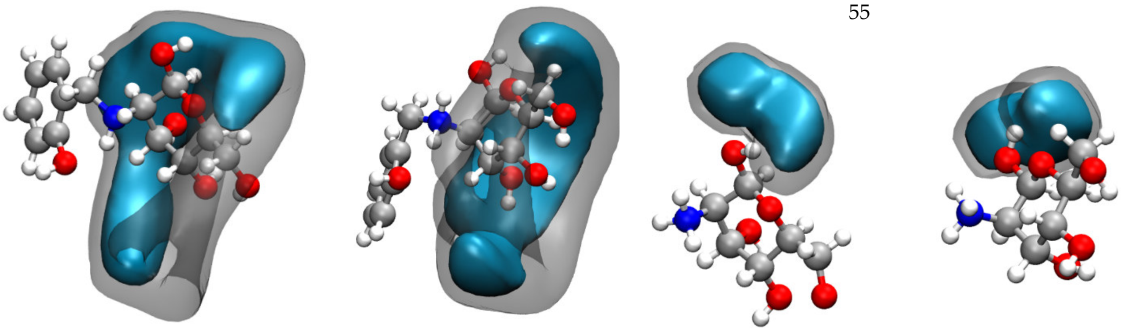

Figure S7. Spatial distribution functions for MD simulations of Cu(II) with: (**A**) and (**B**) RCD+; and (**C**) and (**D**) Chit+. The particle density along the gray isosurface (blue) is 2 nm^{-3}.

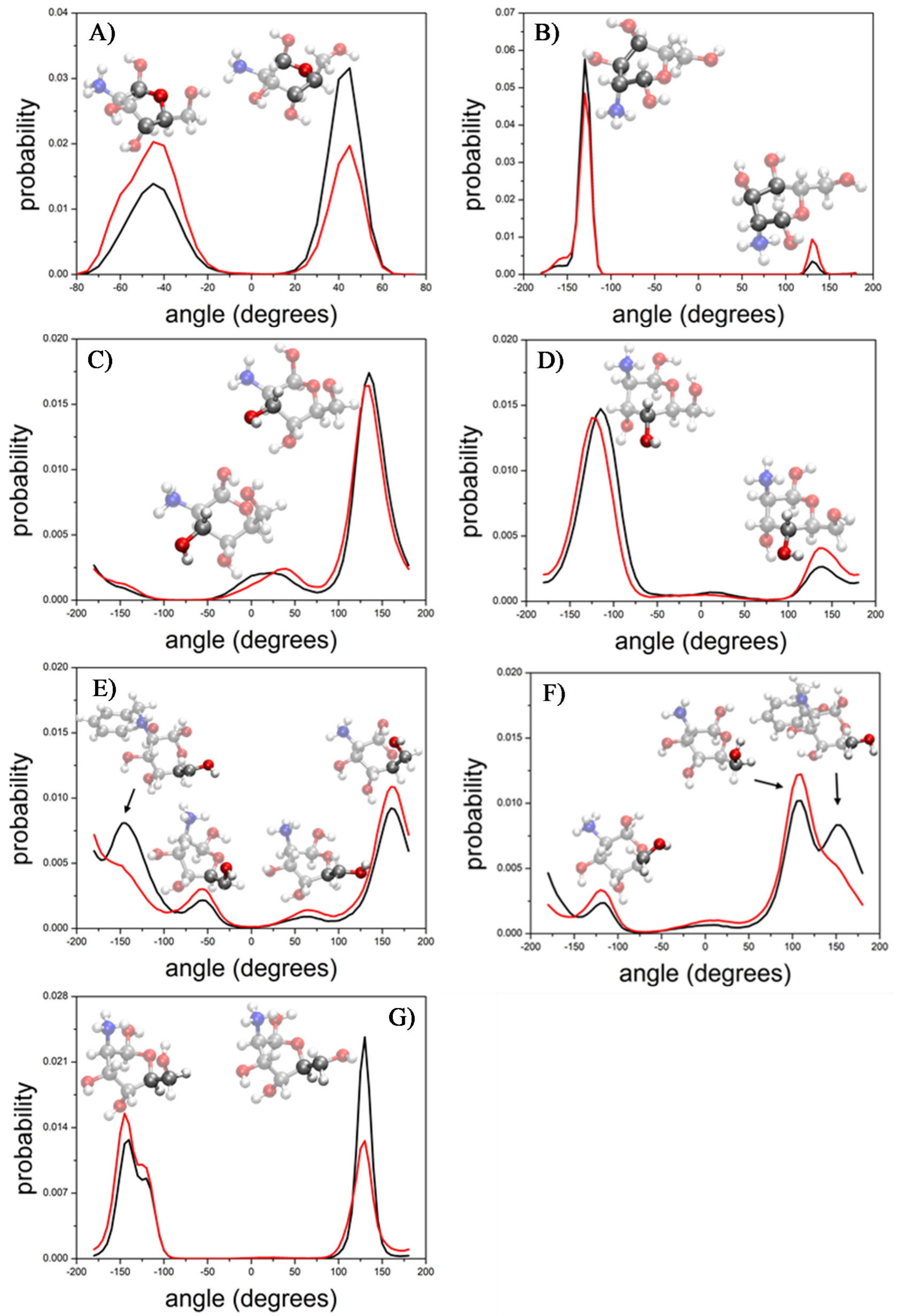

Figure S8. (**A**)-(**G**) Distributions of representative dihedral angles of the Chit+ (red curves) and RCD+ (black curves) structures. The inserted structures are the most likely ones. The atoms defining a specific dihedral angle are emphasized in the corresponding inserted structures.

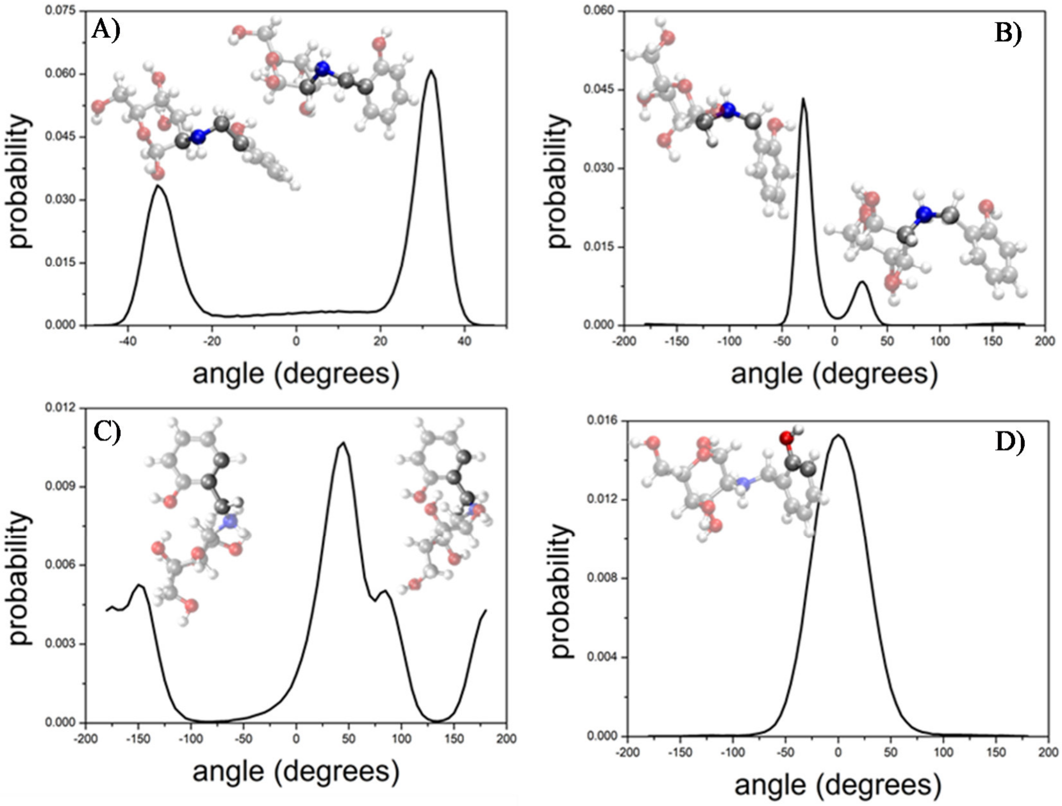

Figure S9. (**A**)-(**D**) distributions of representative dihedral angles of the RCD+ structure. The inserted structures are the most likely ones. The atoms defining a specific dihedral angle are emphasized in the corresponding inserted structures.

Table S1. Fitting parameters of different models of isotherms for Cu(II) adsorption.

Adsorption isotherms			
**Model**	**Parameters**	**RCD3**	**Chitosan**
Langmuir $q_\mathrm{e} = \frac{q_\mathrm{m} K_\mathrm{L} C_\mathrm{e}}{1 + K_\mathrm{L} C_\mathrm{e}}$	q_m / (mg g^{-1})	50 (3)	54 (2)
	K_L / (L mg^{-1})	0.12 (0.05)	0.041 (0.005)
	R^2	0.9031	0.9866
	AIC	18.3	9.6
Langmuir-Freundlich $q_e = \frac{q_\mathrm{m}(K_\mathrm{LF} C_\mathrm{e})^b}{1 + (K_\mathrm{LF} C_\mathrm{e})^b}$	q_m / (mg g^{-1})	78 (11)	53 (3)
	K_LF / (L mg^{-1})$^{1/b}$	0.02 (0.01)	0.042 (0.006)
	b	0.38 (0.04)	1.0 (0.1)
	R^2	0.9928	0.9900
	AIC	−4.7	11.7

Table S2. Fitting parameters of different kinetic models for Cu(II) adsorption.

Adsorption kinetics			
**Model**	**Parameters**	**RCD3**	**Chitosan**
Pseudo-first order $q_t = q_\mathrm{e}(1 - \mathrm{e}^{-k_1 t})$	q_e / (mg g^{-1})	16.4 (0.2)	15.4 (0.3)
	k_1/ (min^{-1})	0.0062 (0.0003)	0.011 (0.001)
	R^2	0.9927	0.9602
	AIC	−4.6	2.2
Pseudo-second order $q_t = \frac{k_2 q_\mathrm{e}^2 t}{1 + k_2 q_\mathrm{e} t}$	q_e / (mg g^{-1})	18.6 (0.2)	16.7 (0.2)
	k_2/ (g mg^{-1} min^{-1})	0.00043 (0.00003)	0.00096 (0.00007)
	R^2	0.9963	0.9942
	AIC	−8.6	-9.9
